# Deep Learning-Based Diagnosis of Epithelial Ovarian Cancer from Whole-Slide Histopathology Images

**DOI:** 10.3390/diagnostics16101470

**Published:** 2026-05-12

**Authors:** Jihyun Chun, Haeyoun Kang, Heewon Chung, Jae-Myung Jang, Jangwon Seo, Taegyu Kim, Woohyun Lee, Cheolhong Park, Mingi Hong, Han-Mac Brian Kim, Messi H. J. Lee, Kyongseok Jang, Chan Kwon Jung, Sang Wun Kim, Ahwon Lee

**Affiliations:** 1 Department of Hospital Pathology, Seoul St. Mary’s Hospital, College of Medicine, The Catholic University of Korea, Seoul 06591, Republic of Korea; hijhchun92@cmcnu.or.kr (J.C.); ckjung@catholic.ac.kr (C.K.J.); 2 Department of Pathology, CHA Bundang Medical Center, CHA University School of Medicine, Seongnam 13496, Republic of Korea; hykang@cha.ac.kr; 3 AI Healthcare Innovation Team, MTS Company Inc., Seoul 06178, Republic of Koreajmjang@mtsco.co.kr (J.-M.J.); lwh1021@mtsco.co.kr (W.L.);; 4 Cancer Research Institute, The Catholic University of Korea, Seoul 06591, Republic of Korea; 5 Department of Obstetrics and Gynecology, Institute of Women’s Life Medical Science, Yonsei University College of Medicine, Seoul 03722, Republic of Korea

**Keywords:** ovary, deep learning, digital pathology, computer-assisted diagnosis, computational pathology

## Abstract

**Background/Objectives**: Ovarian epithelial cancers (EOCs) comprise heterogeneous subtypes with distinct clinical outcomes, making accurate histological subtyping essential for prognosis and treatment planning. Although deep learning using digitized hematoxylin and eosin (H&E) whole-slide images (WSIs) is now widely used, its application to ovarian cancer diagnosis remains limited. **Methods**: In this multicenter study, we analyzed 319 H&E-stained slides from 152 patients with surgically resected EOC. An attention-based multiple instance learning (MIL) framework built on a pathology-specific foundation model (UNI) was used. WSIs were divided into 512 × 512-pixel patches at 40× magnification, and slide-level classification were generated through attention-based aggregation of patch-level feature, followed by patient-level prediction. External validation was performed specifically on the high-grade serous carcinoma (HGSC) cases from The Cancer Genome Atlas (TCGA) dataset. **Results**: The model achieved strong performance, with slide-level and patient-level accuracies of 0.918 and 0.900, respectively, on the test set. In five-fold cross-validation, the mean slide-level AUC was 0.990 (95% CI: 0.983–0.997), and the patient-level AUC was 0.993 (95% CI: 0.989–0.996), indicating consistent results. External validation on TCGA HGSC cases showed robust generalizability, with slide-level and patient-level accuracies of 0.794 and 0.898. F1-scores ranged from 0.832 to 1.000 at the slide-level and from 0.831 to 0.966 at the patient-level, with particularly strong performance for HGSC and clear-cell carcinoma. **Conclusions**: These findings demonstrate the feasibility of deep learning-based models for histological subtyping of EOC using H&E-stained WSIs. This approach may help pathologists achieve more accurate and consistent histological diagnoses of EOC.

## 1. Introduction

Epithelial ovarian cancer (EOC) represents the most prevalent type of gynecologic malignancy [1]. It encompasses a diverse spectrum of tumors with distinct histologic subtypes, including serous, endometrioid, clear-cell, and mucinous carcinomas. These subtypes exhibit substantial differences in their molecular characteristics, clinical presentation, therapeutic response, and overall prognosis [2]. Among them, high-grade serous carcinoma (HGSC) is the most common type of EOC, accounting for approximately 75% of EOC cases. It is characterized by rapid progression and poor prognosis, with an approximate five-year survival rate of 25% [3]. In contrast, low-grade serous carcinoma (LGSC) and mucinous carcinoma generally demonstrate more favorable outcomes, with five-year survival rates of around 85% and 90%, respectively, particularly when detected at an early stage [4,5,6]. Therefore, precise histological subtyping is critical for predicting clinical outcomes and guiding treatment planning.

The diagnosis of EOC is primarily based on histopathological assessment of hematoxylin and eosin (H&E)-stained tissue sections by pathologists. However, distinguishing between histological subtypes can be challenging due to the broad spectrum of morphological features and their frequent overlap [7,8]. In some cases, even immunohistochemical analysis may also produce inconclusive results, further complicating the diagnostic process [9]. These challenges highlight the need for adjunctive tools to improve the accuracy and reproducibility of histological classification. Recently, deep learning (DL)-based image analysis has emerged as a promising strategy to support histopathological diagnosis [10,11]. In the context of EOC, such models may enhance the diagnostic resolution in morphologically ambiguous cases and facilitate more accurate subtype classification.

The recent approval of whole-slide images (WSIs) for primary diagnostic applications has accelerated the integration of digital pathology into routine clinical practice [12]. This transition has enabled computer-aided analysis of H&E-stained slides, which are routinely available for most cancer patients. DL models, particularly those based on convolutional neural networks (CNNs), have shown strong performance in extracting informative features from large-scale histopathological datasets [13,14]. These approaches not only enhance diagnostic accuracy and efficiency but also enable the prediction of histologic subtypes and underlying molecular alterations [15]. As a result, DL provides a cost-effective means to improve the precision and reproducibility of pathological assessments, especially in morphologically heterogeneous cancers such as EOC.

Although recent DL models have shown promise in EOC classification, several important limitations remain. First, traditional DL approaches typically require large number of annotated slides to achieve stable and reliable performance. Second, many existing models have focused primarily on major subtypes, such as HGSC, with less attention given to rarer but clinically meaningful subtypes [16,17,18,19,20]. Third, most previous studies have performed analyses at the slide-level, which does not fully reflect real-world clinical practice, where pathologists integrate information from multiple slides to reach a final diagnosis [16,17,18,19,20]. Finally, previous studies have generally examined images at a single magnification, most commonly either 20× or 40×, limiting direct comparison between resolutions and the ability to determine the most appropriate magnification for accurate subtype classification [16,17,18,19,20].

To address these limitations, this study aims to develop a comprehensive and clinically practical DL model for histological subtyping of EOC using H&E-stained WSIs. Instead of relying heavily on large-scale datasets, we employ Universal Histopathology Model (UNI), a pathology-specific pre-trained foundation model known for maintaining strong performance even with a relatively limited number of training slides [21,22]. By integrating this data-efficient approach within a multiple instance learning (MIL) framework, the model can classify not only the major EOC subtypes but also less common histologic subtypes. In addition, the analysis was extended to the patient level, and performance was directly compared across 20× and 40× magnifications to better reflect clinical workflows. Overall, this approach aims to improve the accuracy and consistency of EOC subtyping in routine pathology practice.

## 2. Materials and Methods

### 2.1. Datasets

This study was approved by the institutional review board of Severance Hospital (IRB no. 4-2023-0062). In this retrospective study, the requirement for informed consent was waived, and no compensation was provided to the participants. All included patients were biologically confirmed as female. A total of 152 patients who underwent surgical resection for ovarian cancer between 2015 and 2024 were included from Severance Hospital, Seoul St. Mary’s Hospital, Samsung Medical Center, and Korea University Anam Hospital in Seoul, Republic of Korea. Among these, only patients with a final diagnosis of EOC were selected for analysis. In addition, normal ovarian tissue samples were obtained from 20 randomly selected patients who underwent hysterectomy at Seoul St. Mary’s Hospital between January and December 2023.

### 2.2. Slide Preparation and Preprocessing

All H&E-stained slides were prepared from surgically resected specimens using formalin-fixed, paraffin-embedded (FFPE) tissue blocks. For histologic subtyping, all WSIs were retrospectively reviewed and the ground truth was established by two pathologists (A. Lee and J. Chun). Any discrepancies in diagnosis were resolved through consensus. For each patient, representative slides with high tumor cellularity were selected, with a median of 58 slides (range, 36–64). All slides were digitized using a Hamamatsu NanoZoomer S360 scanner (Hamamatsu Photonics K.K., Hamamatsu, Japan) at magnifications of 20× and 40×. Slides lacking viable tumor tissue, exhibiting poor image quality, or containing significant artifacts were excluded.

For deep learning analysis, each WSI was divided into non-overlapping patches of 512 × 512 pixels. This patch size was chosen to ensure a consistent input resolution for the foundation model while preserving sufficient cytologic and architectural information. All patches were extracted using a non-overlapping grid, with the stride set equal to the patch size. For multiscale analysis, patches were generated at two magnifications. At 40× magnification, 512 × 512 pixel patches (covering approximately 128 μm × 128 μm) were directly extracted. At 20× magnification, patches were initially extracted at 1024 × 1024 pixels and then downsampled to 512 × 512 pixels to match the input requirements of the feature extractor.

To ensure data quality, a three-stage filtering pipeline was applied. First, tissue regions were identified using Otsu’s thresholding on images downsampled to one-tenth of the original resolution, and patches were extracted only from areas where tissue occupied at least 10% of the patch. Second, white background regions were removed when RGB pixel values exceeded 220, and additional artifacts such as bubbles or overexposed areas were excluded using an HSV saturation threshold. Finally, out-of-focus patches were excluded based on blur detection using the variance of the Laplacian, with only patches meeting the predefined sharpness criteria retained. The resulting high-quality patches at multiple resolutions were then used in the subsequent hierarchical MIL framework.

### 2.3. Deep Learning Model Development

A deep learning-based diagnostic classifier for histopathological WSIs was developed using a MIL framework based on the MILNet architecture. The model comprised three main components: a foundation model-based feature extractor, an attention-based feature aggregator, and a slide-level classifier. These components were further extended to generate patient-level predictions.

To extract features effectively, we employed the UNI, which is a foundation model specifically designed for pathology. This model has been pretrained on a vast dataset, comprising over 100 million histopathology patches and 100,000 WSIs. Within our workflow, we utilized UNI to create compact feature embeddings that are semantically rich, derived from each patch containing tissue samples. These embeddings, gathered at the patch-level, were then introduced into our attention-based MIL framework. By taking advantage of the pretrained representations generated by UNI, we were able to capture biologically significant features without needing any additional annotation or extensive retraining of the model. Specifically, the UNI feature extractor was kept frozen during training, and only the attention-based aggregation module (approximately 344,000 parameters) was trained.

The features extracted from each patch were combined using an attention-based pooling module that assigned adaptive weights to individual patches, reflecting their importance in relation to the overall diagnosis. This process resulted in a single bag-level representation for each slide, summarizing the attention-weighted features effectively.

The attention-based aggregation was implemented using the gated attention mechanism proposed by Ilse et al. [23]. This framework follows a weakly supervised MIL setting, where the model was trained using only slide- or patient-level diagnostic labels without requiring patch-level annotations. In this formulation, each patch was treated as an instance, whereas each slide (or patient) was regarded as a bag.

For each patch i, an unnormalized attention score was computed as:

a_i = w^T (tanh(W_V h_i) ⊙ σ(W_U h_i))

where h_i denotes the feature vector of patch i extracted by UNI, W_V and W_U are learnable weight matrices, ⊙ represents element-wise multiplication, and w is a learnable projection vector.

The attention scores were normalized using the softmax function to obtain attention weights α_i, which were then used to generate the bag-level representation z:

α_i = exp(a_i)/Σ_j exp(a_j),  z = Σ_i α_i h_i

The attention weight α_i reflects the relative diagnostic relevance of each patch, allowing the model to identify informative regions that can be visualized as attention heatmaps. The aggregated representation z summarizes the entire slide into a single feature vector, which was subsequently passed to the classification layer.

For patient-level prediction, the same attention mechanism was applied hierarchically across two levels.

First, patch-level features within each slide were aggregated into a slide-level representation z_s:

z_s = Σ_i α_i^(s) h_i

(Level 1: Patch → Slide)

Second, the slide-level representations were further aggregated using a separate gated attention module to obtain the patient-level representation z_pat:

z_pat = Σ_s β_s z_s

(Level 2: Slide → Patient)

The final patient-level prediction was obtained as:

ŷ = softmax(FC(z_pat))

where β_s denotes the attention weight assigned to slide s, representing its contribution to the final patient-level prediction.

This aggregated representation was passed to a fully connected classification layer within the MILNet architecture, producing slide-level probabilities for the predefined diagnostic subtypes. For patient-level prediction, we used a two-level hierarchical gated attention-based aggregation. First, patch-level embeddings within each slide were aggregated to form a slide-level representation. These slide-level representations were then combined using learned attention weights to generate a patient-level embedding, which was finally passed to a fully connected layer for classification. The total cohort of 152 patients was randomly split into training, validation, and testing sets in an 80:10:10 ratio. To ensure rigorous evaluation and prevent data leakage, both the dataset split and five-fold cross-validation were performed at the patient-level rather than the slide-level. Specifically, all WSIs from the same patient were assigned to the same fold. This patient-level split was consistently applied to both the slide-level and patient-level models. The slide-level ABMIL model was optimized using the Adam optimizer with a learning rate of 1 × 10^−5^. The patient-level hierarchical model was trained using Adam with an initial learning rate of 1 × 10^−4^ and a cosine annealing warm restart schedule (T_0_ = 20, T_mult = 2). Training was run for up to 600 epochs. To prevent overfitting, early stopping based on validation AUC was applied with a patience of 15 epochs and a minimum change threshold (δ = 0.001). Depending on the subtype and magnification, early stopping typically occurred between 100 and 200 epochs (Figure S1). To improve interpretability, we retained the attention scores from the MIL module and visualized them to emphasize the patches that played the most crucial role in the slide-level predictions. The final classification was achieved by choosing the diagnostic class that had the highest predicted probability based on the output from the MILNet.

### 2.4. High-Score Patch Extraction and Clustering Analysis

To investigate morphological patterns most strongly associated with the model’s predictions, clustering analysis was performed on high-attention patches. Attention scores were extracted from the MIL attention module and normalized using min–max scaling. From each WSI, the top-k patches with the highest attention scores were selected for subsequent analysis.

For each selected patch, a one-dimensional feature vector was obtained from the UNI encoder by applying global average pooling to the final layer of the model. These feature vectors were dimensionally reduced using the Principal Component Analysis (PCA) algorithm to allow for visualization in two dimensions. Clustering was performed using a Gaussian Mixture Model (GMM), and the optimal number of clusters (k) was determined using the Elbow method based on the Within-Cluster Sum of Squares (WCSS) values.

### 2.5. External Validation Using TCGA Databases

For external validation, we used H&E-stained WSIs from primary tumor specimens in The Cancer Genome Atlas (TCGA)-OV project. Since this dataset is composed mainly of HGSC cases and all slides are scanned at 20× magnification, the analysis was limited to the HGSC subtype using the 20× model. During preprocessing, slides that could not be read with the OpenSlide library were excluded. To mitigate potential domain shift, we applied RandStainNA as a stain augmentation method during training [24]. All subsequent evaluations, including slide-level and patient-level accuracy, were conducted in Python using scikit-learn (v3.8.20).

### 2.6. Visualization and Statistics

To visualize diagnostically relevant regions, attention score heatmaps were overlaid on the WSIs using a color map (Figure S2). For each WSI, the overall classification outcome was determined by averaging the predicted probabilities across individual patches. Classifier performance was assessed using receiver operating characteristic (ROC) curves and the corresponding area under the curve (AUC). Model performance was quantitatively evaluated on training, validation, and test sets using five-fold cross-validation. For each diagnostic class, metrics including precision (PPV), recall (sensitivity), specificity, negative predictive value (NPV), and F1-score were calculated. Confusion matrices were generated to examine class-wise misclassification patterns. Overall accuracy and cross-entropy loss were also reported.

All analyses were conducted using Python (scikit-learn v3.8.20). A *p*-value of < 0.05 was considered statistically significant.

## 3. Results

### 3.1. Cohort Characteristics

The distribution of histological subtypes and the number of WSIs per subtype are summarized in Table 1. The patients were classified into nine subtypes according to the fifth edition of the World Health Organization (WHO) classification [25], including HGSC, LGSC, clear-cell carcinoma, endometrioid carcinoma, mucinous carcinoma, malignant Brenner tumor, mesonephric-like/mesonephric adenocarcinoma, undifferentiated/dedifferentiated carcinoma, and carcinosarcoma. Among these, malignant Brenner tumors, mesonephric-like/mesonephric adenocarcinoma, undifferentiated/dedifferentiated carcinoma, and carcinosarcoma are relatively uncommon, with fewer cases than other subtypes. Therefore, they were grouped together and classified as “rare-type carcinomas.” Of the nine histological subtypes, HGSC was the most common subtype in the cohort (*n* = 50).

### 3.2. MIL Model

**Figure 1:**
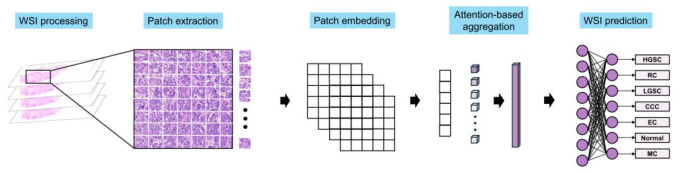
Overview of the proposed attention-based multiple instance learning (MIL) model. Whole-slide images were scanned at 20× and 40× magnifications and divided into non-overlapping patches. Feature embeddings were extracted from each patch using a pre-trained UNI model and aggregated using an attention-based MIL framework (MILNet). Patch-level features were hierarchically aggregated to generate slide-level and patient-level predictions for histologic subtypes.

An overview of the proposed attention-based MIL model based on MILNet is shown in Figure 1. For each patch, feature vectors were extracted using a pre-trained UNI and used as input to attention-based MIL models trained separately at 20× and 40× magnifications. Slide-level predictions were computed separately for each magnification. Patient-level predictions were then obtained using a two-level hierarchical gated attention-based aggregation. At the first level, patch-level embeddings within each slide were aggregated into a slide-level representation. At the second level, these slide-level representations were combined using learned attention weights to form a patient-level embedding, which was then passed to a fully connected classification layer.

**Figure 2:**
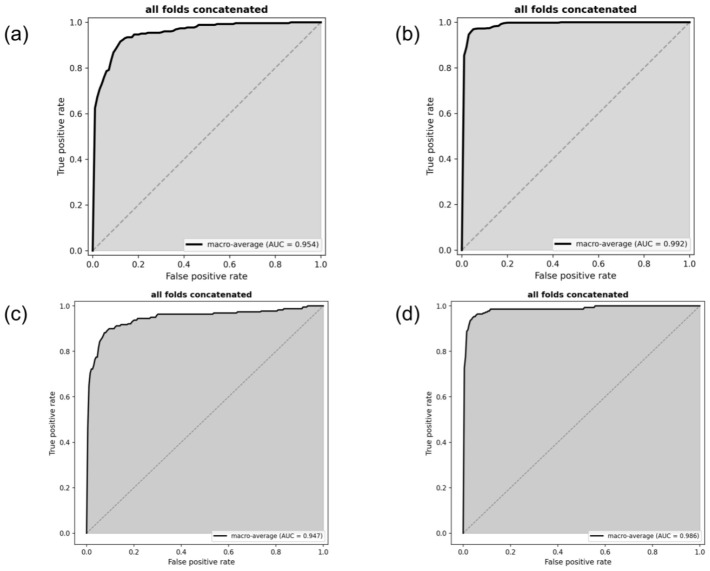
Slide-level and patient-level classification results for the 20× and 40× magnification models. Receiver operating characteristic (ROC) curves representing the concatenated results of all five cross-validation folds are shown to evaluate the overall performance of the models. The panels display the ROC curves for (**a**) slide-level predictions at 20× magnification, (**b**) slide-level predictions at 40× magnification, (**c**) patient-level predictions at 20× magnification, and (**d**) patient-level predictions at 40× magnification.

Five-fold cross-validation was used for training and internal validation. In validation, the ROC curves for the 20× and 40× magnification models are presented in Figure 2. At the slide-level, the mean AUC-ROC across seven histologic subtypes was 0.951 (95% CI, 0.909–0.994) for the 20× model and 0.990 (95% CI, 0.983–0.997) for the 40× model (Table 2). At the patient-level, the 20× model achieved an AUC-ROC of 0.947 (95% CI, 0.921–0.969) with an accuracy of 0.951 ± 0.027. The 40× model showed a higher AUC-ROC of 0.993 (95% CI, 0.989–0.996), although with a slightly lower accuracy of 0.900 ± 0.029 (Table 3). Overall, the 40× model demonstrated superior discriminative performance.

### 3.3. Quantitative Evaluation in the Classification of EOC Subtypes

In the slide-level evaluation on the test set, clear-cell and rare-type carcinomas achieved the highest F1-scores at 40× magnification (both 1.000 ± 0.000, Table S1). At 20× magnifications, clear-cell carcinoma showed the highest F1-scores (0.865 ± 0.067, Table S2). In contrast, mucinous carcinoma had the lowest F1-score at 40×, whereas HGSC showed the lowest performance at 20× (0.832 ± 0.017 and 0.342 ± 0.249, respectively). The overall loss was 0.331 ± 0.070 at 40× and 0.793 ± 0.161 at 20×, indicating stable model convergence (Table 2). During internal validation, accuracies were 0.886 ± 0.045 at 40× and 0.708 ± 0.117 at 20×, which were comparable to those observed in the test set.

Similarly, in the patient-level evaluation, clear-cell carcinoma achieved the highest F1-score at both magnifications, with values of 0.966 ± 0.046 at 40× (Table S3) and 0.903 ± 0.092 at 20× (Table S4). In contrast, LGSC showed the lowest F1-scores at both 40× and 20× (0.831 ± 0.104 and 0.562 ± 0.140, respectively). The overall patient-level loss was 0.322 ± 0.203 at 40× and 0.465 ± 0.627 at 20× (Table 3). During internal validation, accuracies were 0.921 ± 0.031 at 40× and 0.787 ± 0.095 at 20×. The 40× model showed consistent performance across datasets, whereas the 20× model demonstrated lower accuracy during internal validation compared with the patient-level test set.

### 3.4. Histopathological Interpretation of Model Predictions

**Figure 3:**
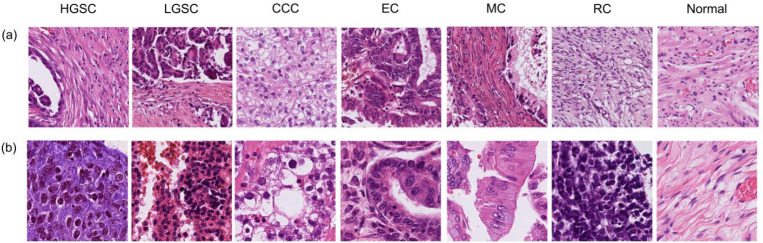
Representative images of the top five high-attention patches across epithelial ovarian cancer subtypes at (**a**) 20× and (**b**) 40× magnifications. For each histologic subtype, representative images selected from the top five patches with the highest attention scores are shown, highlighting regions that most strongly contributed to the model’s classification. Abbreviations: HGSC, high-grade serous carcinoma; LGSC, low-grade serous carcinoma; CCC, clear-cell carcinoma; EC, endometrioid carcinoma; MC, mucinous carcinoma; RC, rare-type carcinoma.

To investigate histological features contributing to the model’s predictions, high-attention patches were analyzed. Patches with the highest attention scores, indicating the greatest contribution to classification, were extracted from representative cases in the cohort. Representative images corresponding to the top five attention scores for each EOC subtype are shown in Figure 3.

The representative patches assigned to each subtype exhibited distinct histological features. Patches classified as HGSC were characterized by marked nuclear pleomorphism, prominent nucleoli, and papillary architecture. Those classified as LGSC predominantly showed uniform small nuclei and papillary structures with fibrovascular cores. Patches assigned to clear-cell carcinoma demonstrated abundant clear to eosinophilic cytoplasm, whereas those classified as endometrioid carcinoma exhibited glandular architecture with columnar cell morphology. Mucinous carcinoma patches were characterized by abundant mucinous cytoplasm. Furthermore, features extracted at 40× magnification showed better concordance with established histopathologic characteristics, particularly for HGSC, LGSC, and rare-type carcinomas. When reviewing the top five high-attention patches, the 20× model frequently highlighted non-informative stromal regions, whereas the 40× model more consistently focused on diagnostically relevant cellular details (Figure S3). These findings indicate that the model’s decision-making process is closely aligned with established histopathological hallmarks of EOC subtypes. Notably, compared with the 20×, the 40× model showed better agreement with known histologic features and more consistently focused on diagnostically relevant tumor regions. This consistency enhances the interpretability of the model and supports the biological plausibility of the classification results.

### 3.5. Subtype Misclassification Analysis

**Figure 4:**
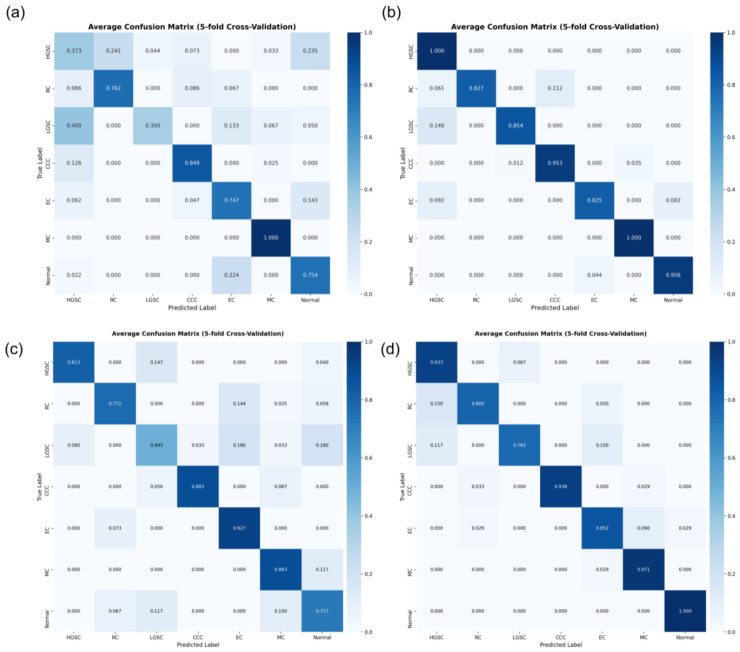
Confusion matrices showing the classification performance of the 20× and 40× models across seven histologic subtypes of epithelial ovarian cancer at (**a**) slide-level 20×, (**b**) slide-level 40×, (**c**) patient-level 20×, and (**d**) patient-level 40× magnifications. The 40× models demonstrated overall higher accuracy at both levels, correctly identifying the vast majority of HGSC, MC, and CCC cases, while noticeable misclassifications were mainly observed in RC, EC, and LGSC. Abbreviations: HGSC, high-grade serous carcinoma; LGSC, low-grade serous carcinoma; CCC, clear-cell carcinoma; EC, endometrioid carcinoma; MC, mucinous carcinoma; RC, rare-type carcinoma.

Slide- and patient-level confusion matrices were constructed to evaluate classification performance across EOC subtypes (Figure 4). Overall, the performance was consistently better at 40× than 20× across both evaluation levels.

At the slide-level, the model correctly identified most HGSC (100%) and mucinous carcinoma (100%) cases, consistent with their relatively high F1-scores at 40× magnification. In contrast, endometrioid carcinoma showed the greatest confusion at 40×, with the majority of its misclassifications (9.2%) occurring as HGSC, reflecting the overlapping histological features between these subtypes. Similarly, LGSC also exhibited a considerable degree of misclassification, with 14.6% of cases predicted as HGSC, likely due to their shared architectural characteristics.

At the patient-level, the 40× model correctly identified the majority of mucinous carcinoma (97.1%), clear-cell carcinoma (93.8%), and HGSC (93.3%) cases. In contrast, LGSC and rare-type carcinomas showed the highest rates of misclassification. Specifically, 15.0% of rare-type carcinoma cases were misclassified as HGSC, likely because many rare-types exhibit high-grade nuclear features similar to HGSC. Consistent with the slide-level findings, LGSC was frequently misclassified as HGSC (11.7%) and endometrioid carcinoma (10.0%).

To further investigate the diagnostic basis behind these errors, we visualized representative misclassified slides and their corresponding attention heatmaps (Figure S4). For example, a clear-cell carcinoma case misclassified as HGSC at 20× magnification showed that the model correctly focused on relevant tumor regions but appeared to misinterpret shared solid growth patterns and high-grade nuclear features as those of HGSC. At 40× magnification, a mucinous carcinoma slide was misclassified as a rare-type carcinoma. This is likely related to the morphological overlap among rare carcinoma subtypes, including malignant Brenner tumors, mesonephric-like adenocarcinoma, mesonephric adenocarcinoma, undifferentiated carcinoma, and dedifferentiated carcinoma, many of which can show overlapping nuclear features. In particular, mesonephric and mesonephric-like adenocarcinomas may contain eosinophilic intraluminal material, which can also be seen in mucinous carcinoma. The attention heatmap indicated that the model focused on these intraluminal eosinophilic materials, suggesting that it captured diagnostically challenging mimics that are also well recognized pitfalls in routine pathological diagnosis.

Collectively, these findings provide a more detailed view of classification errors at both slide- and patient-levels, highlighting subtypes where model performance could be further improved.

### 3.6. External Validation on the TCGA Database

To further assess how well our model generalizes, we conducted external validation using TCGA database. Because the publicly available ovarian cancer dataset (TCGA-OV) is largely composed of HGSC cases and provides WSIs only at 20× magnification, we focused this analysis on the HGSC subtype using the 20× model. In total, the external validation set included 167,621 patches extracted from 467 H&E-stained slides representing 196 patients.

The model showed stable and reliable performance on this independent cohort. At the slide-level, the 20× model achieved an accuracy of 0.794 (95% CI: 0.755–0.829). When predictions were aggregated at the patient-level, performance improved noticeably, with accuracy increasing to 0.898 (95% CI: 0.848–0.933). Together, these findings support the robustness and generalizability of our model for identifying the HGSC subtype in a well-established external dataset.

## 4. Discussion

In this study, a DL model was developed for histological subtyping of EOC using WSIs. The attention-based MIL framework demonstrated strong performance at both the slide- and patient-levels, with superior results observed at 40× magnification. High-attention patches corresponded well with established histopathological features, supporting the interpretability of the model and its potential to enhance diagnostic accuracy.

Previous studies have largely focused on binary classification tasks. For example, Abd El-Latif et al. reported a DL model with high accuracy for distinguishing cancer from non-cancer, but their study did not address histological subtypes [18]. Ziyambe et al. likewise focused on a narrower setting, proposing a CNN to separate HGSC from normal tissue, without extending the analysis to multiple subtypes [19]. More recently, efforts have shifted toward subtype classification. Saha et al. demonstrated strong performance, although their analysis was limited to the four major EOC subtypes and did not include LGSC or rarer entities [20]. Breen et al. further advanced this line of work by applying histopathology foundation models to classify the five major subtypes [22]. Despite this methodological progress, their study still focused on the most common categories. In contrast, the present study considers a broader range of categories, including not only the common EOC subtypes but also normal tissue and less frequent histological variants, thereby providing a more comprehensive framework for potential diagnostic use.

Furthermore, our findings highlight the strong data efficiency achieved when using a pathology-specific foundation model. Traditional DL models based on standard backbones such as ResNet or Vision Transformers (ViT) typically require large amounts of annotated data to achieve robust performance. This is largely because models pre-trained on natural images do not transfer well to histopathology due to the substantial domain gap. For instance, a recent comprehensive evaluation by Breen et al. utilized a large training cohort of 1864 WSIs to assess various models for ovarian cancer subtyping [22]. In contrast, our study achieved highly competitive performance using a relatively small dataset of 359 WSIs from 152 patients. This level of data efficiency can be attributed to the use of UNI, a foundation model pre-trained on a large-scale collection of pathology images (over 100 million tissue patches), as our feature extractor. Unlike standard models, UNI is able to capture intricate cytologic and architectural features without requiring extensive fine-tuning. By combining these robust pre-trained features with an attention-based MIL classifier, our approach not only reduces the need for large-scale local datasets and lowers the risk of overfitting, but also provides improved interpretability.

Across both evaluation levels, the model demonstrated strong classification performance for HGSC and clear-cell carcinoma, whereas LGSC and endometrioid carcinoma were more challenging to classify accurately. The relatively lower performance for LGSC likely reflects its inherent histologic ambiguity, as it can be difficult to distinguish on H&E slides even for experienced pathologists [26]. In contrast, HGSC and clear-cell carcinoma, which are morphologically more distinctive and generally easier to recognize [27], were classified with high accuracy. Visualization analysis further showed that high-attention patches corresponded to histologic features consistent with established morphological criteria. In addition, subtype misclassification patterns were primarily observed among histologically similar subtypes, suggesting that the model’s errors may reflect underlying diagnostic ambiguity rather than random misclassification. Taken together, these findings indicate that the model captures key histopathologic features and meaningful morphological differences among EOC subtypes. This approach may support pathologists in achieving more accurate and consistent histologic diagnoses.

In our analysis, the 40× model consistently outperformed the 20× model. Although lower magnifications capture broader tissue architecture [27], this is insufficient for accurate EOC subtyping for several reasons. First, architectural patterns such as solid, papillary, and glandular structures are shared across different subtypes and can also coexist within a single tumor. Second, 20× patches cover larger tissue areas and therefore more often include non-informative regions such as stroma and necrosis. Consistent with this, our review of the top five attention patches showed that 20× patches frequently contained only stromal tissue, whereas this was much less common at 40× magnification. Given this overlap in architecture and the presence of background tissue, accurate subtyping relies more heavily on cytologic-level details. The superior performance of the 40× model supports this, suggesting that detailed cytologic features such as nuclear atypia, chromatin patterns, and cytoplasmic characteristics play a key role in distinguishing EOC subtypes and highlighting the importance of higher-resolution inputs for diagnostic models.

While recent studies have demonstrated the potential of DL models for histological classification of EOC, most have been limited to image- or slide-level predictions [18,19,20]. In clinical practice, however, pathologists do not make diagnoses based on individual slides but instead integrate findings across multiple slides from a single patient. To better reflect this workflow, the present study extended the analysis to patient-level prediction. By demonstrating strong performance at both the slide- and patient-levels, the model more closely aligns with real-world diagnostic processes. This suggests that the proposed approach may offer more practical and clinically relevant support for pathologists. Furthermore, we evaluated the model on an independent external cohort from the TCGA database to assess its robustness and generalizability. Despite potential domain shifts, the 20× model achieved a patient-level accuracy of 0.898 for the HGSC subtype when stain augmentation (RandStainNA) was applied. These results suggest that the foundation model-based approach remains reliable across different institutions and scanning conditions.

This study has several limitations. First, the inclusion of rare-type carcinomas inevitably introduced class imbalance due to their low incidence. To reduce the risk of overfitting, we applied several safeguards, including a frozen foundation model backbone, rigorous patient-level cross-validation, and early stopping. However, despite these efforts, the limited number of cases for rare entities remains an inherent limitation of this study. As a result, the high performance observed in these subtypes may still be overly optimistic and should be interpreted with caution. Further validation using larger, multi-institutional cohorts is therefore required. Second, predictions for rare-type carcinomas should not be regarded as definitive diagnoses. For instance, confirming carcinosarcoma requires careful evaluation of multiple tumor areas to identify both carcinomatous and sarcomatous components. Accordingly, a rare-type prediction by the model should be interpreted as a signal of atypical histologic features that warrants further detailed pathological review. Finally, due to computational constraints and the focus on using a pathology-optimized foundation model, this study did not perform direct comparisons or ablation studies with standard CNN or ViT backbones. We acknowledge that such analyses would be valuable and plan to explore them in future work. Nevertheless, to the best of our knowledge, this is among the first studies to classify a broad range of EOC subtypes, including rare types, based on histopathologic features, highlighting the novelty and potential significance of this approach.

## 5. Conclusions

This study demonstrated that an attention-based MIL model can effectively classify EOC subtypes from H&E-stained WSIs. The model showed strong performance, particularly for HGSC and clear-cell carcinomas, and captured key histopathologic features. This approach may improve the accuracy and reproducibility of histologic diagnosis in EOC.

## Figures and Tables

**Table 1:** Number of patients and whole-slide images by histological subtype of epithelial ovarian cancer in the cohorts.

Subtype		Number of Patients	Number of WSIs	Number of Patches
HGSC		50	58	861,014
LGSC		10	34	364,850
CCC		22	63	864,112
EC		23	64	1,205,189
MC		24	64	976,129
Rare-type carcinoma	MBT	1	2	43,061
	MLA, MA	5	8	193,071
	UDC, DDC	6	7	159,525
	Carcinosarcoma	12	19	498,359
Normal		20	40	909,808

Abbreviations: WSI, Whole-slide images; HGSC, high-grade serous carcinoma; LGSC, low-grade serous carcinoma; CCC, clear-cell carcinoma; EC, endometrioid carcinoma; MC, mucinous carcinoma; MBT, malignant Brenner tumor; MLA, mesonephric-like adenocarcinoma; MA, mesonephric adenocarcinoma; UDC, undifferentiated carcinoma; DDC, dedifferentiated carcinoma.

**Table 2:** Slide-level model performance metrics (accuracy, overall loss, and AUC) in the cohort at 20× and 40× magnifications.

	20×	40×
AUC-ROC	0.951 (0.909–0.994)	0.990 (0.983–0.997)
Accuracy	0.702 ± 0.066	0.918 ± 0.034
Overall loss	0.793 ± 0.161	0.331 ± 0.070

Abbreviations: AUC-ROC, area under the receiver operating characteristic curve. Values in parentheses are 95% CI.

**Table 3:** Patient-level model performance metrics (accuracy, overall loss, and AUC) in the cohort at 20× and 40× magnifications.

	20×	40×
AUC-ROC	0.947 (0.921–0.969)	0.993 (0.989–0.996)
Accuracy	0.951 ± 0.027	0.900 ± 0.029
Overall loss	0.465 ± 0.627	0.322 ± 0.203

Abbreviations: AUC-ROC, area under the receiver operating characteristic curve. Values in parentheses are 95% CI.

## Data Availability

The data presented in this study are available on request from the corresponding author. The data are not publicly available due to privacy and ethical restrictions.

## Supplementary Materials

The following supporting information can be downloaded at https://www.mdpi.com/article/10.3390/diagnostics16101470/s1, Supplementary Figure S1. Average learning curves for the 20× and 40× magnification models; Supplementary Figure S2. Attention score heatmaps across epithelial ovarian cancer subtypes; Supplementary Figure S3. All top five high-attention patches across epithelial ovarian cancer subtypes; Supplementary Figure S4. Representative examples of slide-level misclassifications and attention heatmaps; Supplementary Table S1. Slide-level classification performance of epithelial ovarian cancer subtypes in the cohorts at 40× magnification; Supplementary Table S2. Slide-level classification performance of epithelial ovarian cancer subtypes in the cohorts at 20× magnification; Supplementary Table S3. Patient-level classification performance of epithelial ovarian cancer subtypes in the cohorts at 40× magnification; Supplementary Table S4. Patient-level classification performance of epithelial ovarian cancer subtypes in the cohorts at 20× magnification.

## Abbreviations

The following abbreviations are used in this manuscript:

AUC	Area Under the Curve
CI	Confidence Interval
CNN	Convolutional Neural Network
DL	Deep Learning
EOC	Epithelial Ovarian Cancer
FFPE	Formalin-Fixed Paraffin-Embedded
GMM	Gaussian Mixture Model
H&E	Hematoxylin and Eosin
HGSC	High-Grade Serous Carcinoma
IRB	Institutional Review Board
LGSC	Low-Grade Serous Carcinoma
MIL	Multiple Instance Learning
NPV	Negative Predictive Value
PCA	Principal Component Analysis
PPV	Positive Predictive Value
ROC	Receiver Operating Characteristic
UNI	Universal Histopathology Model
WHO	World Health Organization
WSI	Whole-Slide Image
