# Supplementary material for: Deep Learning-Based Diagnosis of Epithelial Ovarian Cancer from Whole-Slide Histopathology Images
Source: Diagnostics (Basel). 2026 May 12;16(10):1470. doi: 10.3390/diagnostics16101470 (PMC13206691; doi:10.3390/diagnostics16101470)
Supplement: Supplementary file 1 [file diagnostics-16-01470-s001.zip › diagnostics-4292845-supplementary.pdf]

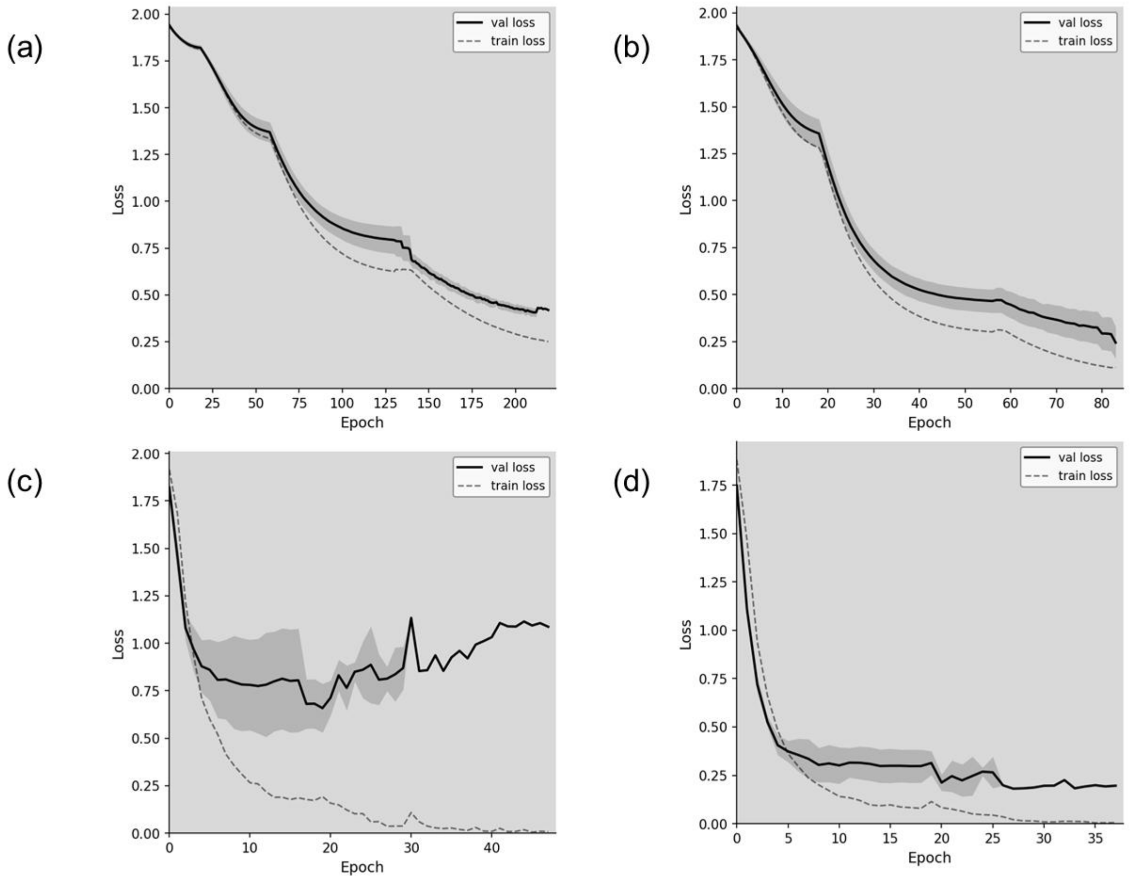

Supplementary Figure S1. Average learning curves for the 20× and 40× magnification models. Training and validation loss curves representing the average results of all five cross-validation folds are shown to evaluate the training stability of the models. The panels display the learning curves for (**a**) the slide-level model at 20× magnification, (**b**) the slide-level model at 40× magnification, (**c**) the patient-level model at 20× magnification, and (**d**) the patient-level model at 40× magnification.

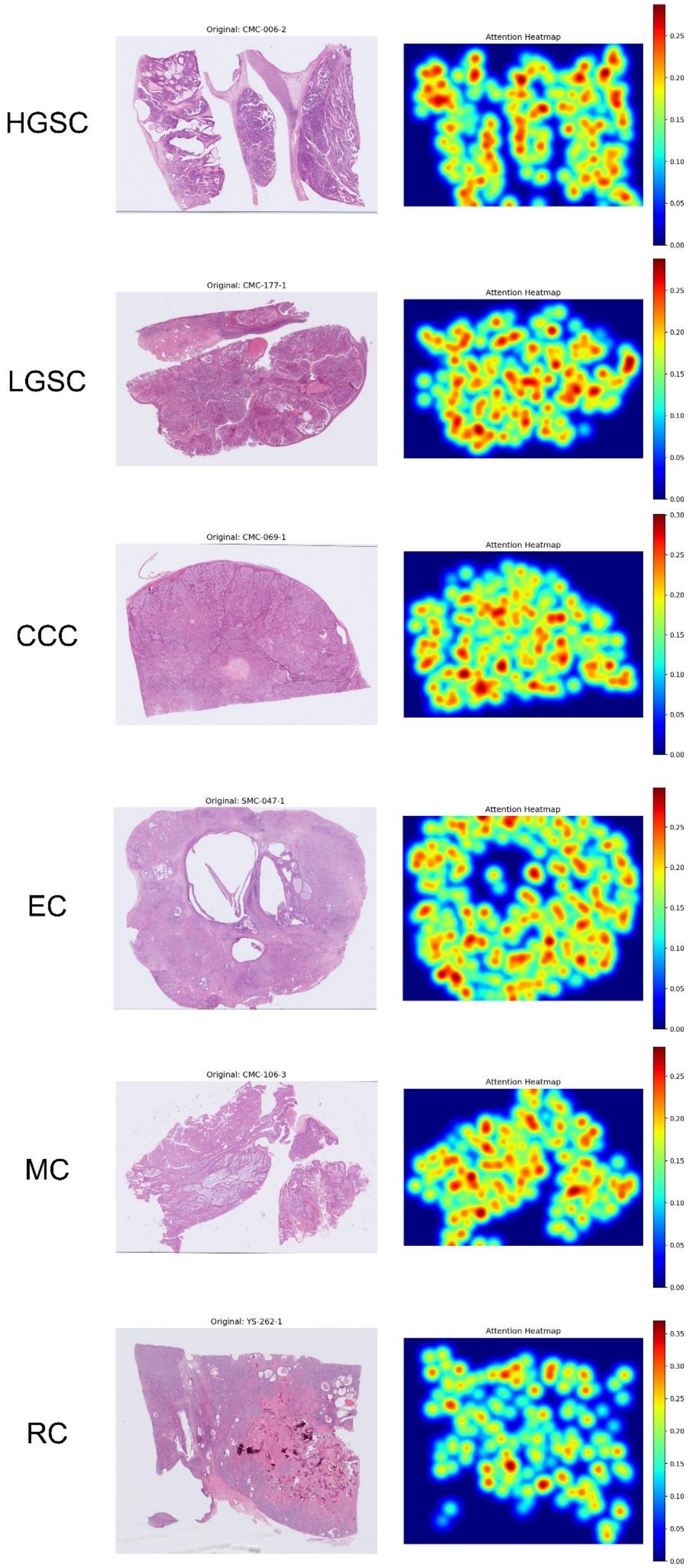
HGSC
Original: CMC-006-2
Attention Heatmap
LGSC
Original: CMC-177-1
Attention Heatmap
CCC
Original: CMC-069-1
Attention Heatmap
EC
Original: SMC-047-1
Attention Heatmap
MC
Original: CMC-106-3
Attention Heatmap
RC
Original: YS-262-1
Attention Heatmap

Supplementary Figure S2. Attention score heatmaps across epithelial ovarian cancer subtypes. Representative original whole-slide images (left) and corresponding attention heatmaps (right) for each subtype. Red regions indicate areas with high diagnostic significance (high attention scores), while blue regions represent low significance. Abbreviations: HGSC, high-grade serous carcinoma; LGSC, low-grade serous carcinoma; CCC, clear-cell carcinoma; EC, endometrioid carcinoma; MC, mucinous carcinoma; RC, rare-type carcinoma.

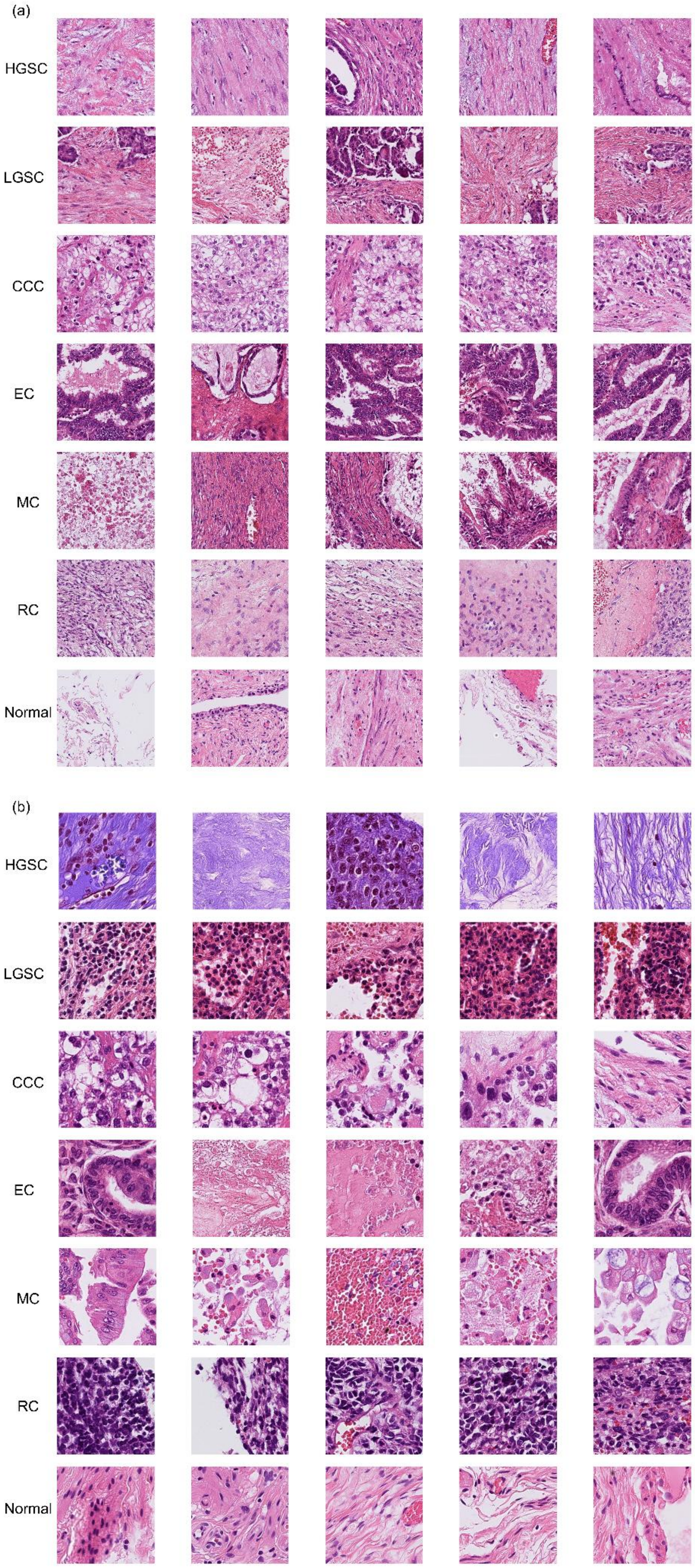
(a)
HGSC
LGSC
CCC
EC
MC
RC
Normal
(b)
HGSC
LGSC
CCC
EC
MC
RC
Normal

Supplementary Figure S3. All top five high-attention patches across epithelial ovarian cancer subtypes. This figure presents the complete set of the top five high-attention patches at (**a**) 20× and (**b**) 40× magnifications for each histologic subtype. Notably, the 20× model frequently highlights non-informative areas, assigning high attention to patches consisting entirely of stroma. In contrast, while such stromal patches are occasionally observed at 40×, this higher magnification more consistently focuses on diagnostic cellular details. Abbreviations: HGSC, high-grade serous carcinoma; LGSC, low-grade serous carcinoma; CCC, clear-cell carcinoma; EC, endometrioid carcinoma; MC, mucinous carcinoma; RC, rare-type carcinoma.

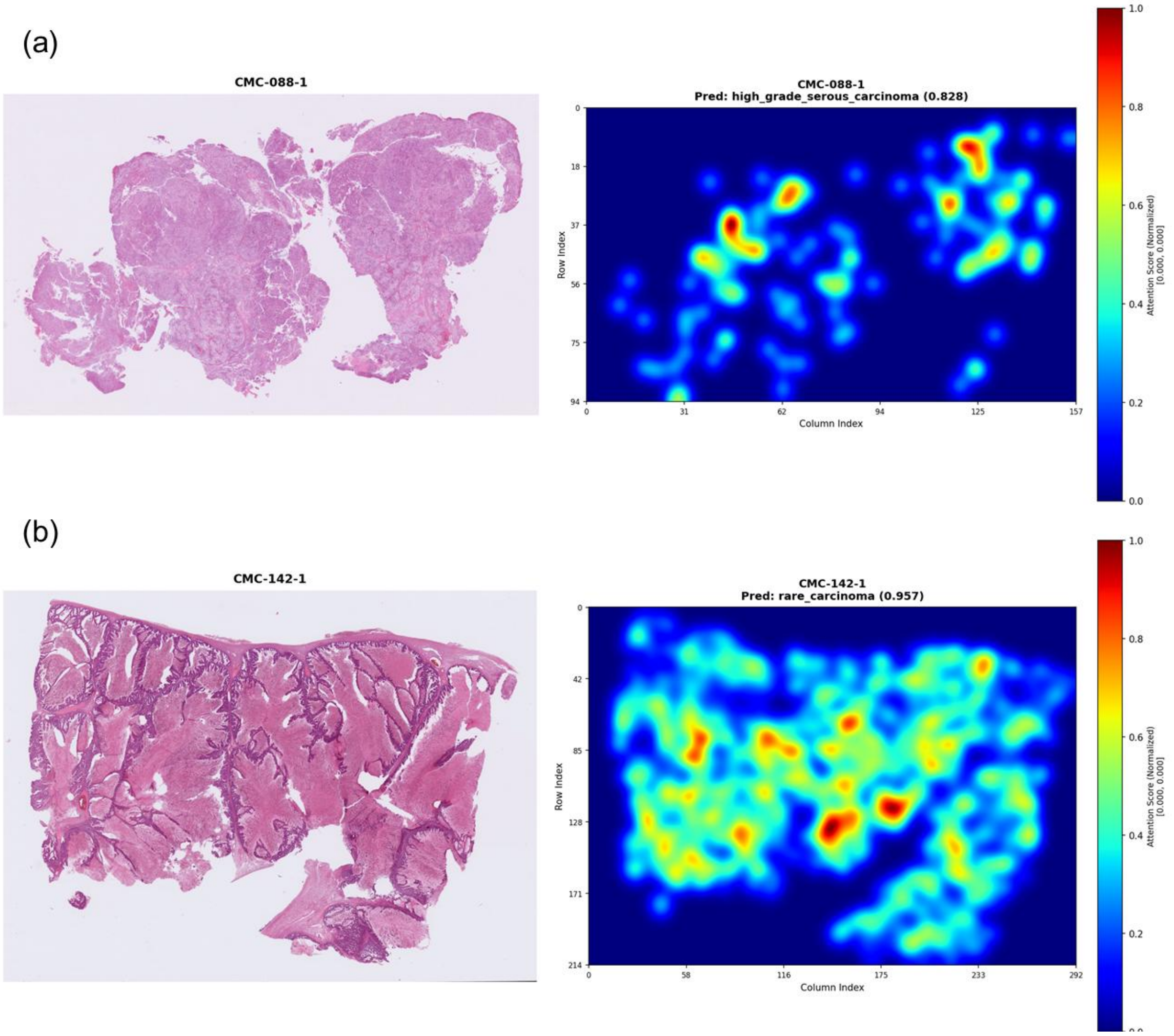

Supplementary Figure S4. Representative examples of slide-level misclassifications and attention heatmaps. This figure presents misclassified slides and their corresponding attention heatmaps at 20× and 40× magnifications. Specifically, (**a**) shows a clear-cell carcinoma misclassified as high-grade serous carcinoma (HGSC) at 20× magnification, where the heatmap focuses on solid growth patterns and high-grade nuclear features mimicking HGSC. In addition, (**b**) illustrates a mucinous carcinoma misclassified as a rare-type carcinoma at 40× magnification, with the heatmap highlighting intraluminal eosinophilic materials and nuclear overlapping that represent complex diagnostic mimics.

Supplementary Table S1. Slide-level classification performance of epithelial ovarian cancer subtypes in the cohorts at 40× magnification.

Subtype	Sensitivity	Specificity	PPV	NPV	F1
HGSC	1.000 ± 0.000	0.970 ± 0.027	0.857 ± 0.062	1.000 ± 0.000	0.923 ± 0.045
LGSC	0.750 ± 0.035	1.000 ± 0.000	1.000 ± 0.000	0.973 ± 0.053	0.857 ± 0.041
CCC	1.000 ± 0.000	1.000 ± 0.000	1.000 ± 0.000	1.000 ± 0.000	1.000 ± 0.000
EC	0.887 ± 0.043	0.970 ± 0.015	0.857 ± 0.086	0.970 ± 0.023	0.857 ± 0.064
MC	0.823 ± 0.011	0.913 ± 0.055	0.806 ± 0.028	0.966 ± 0.083	0.832 ± 0.017
RC	1.000 ± 0.000	1.000 ± 0.000	1.000 ± 0.000	1.000 ± 0.000	1.000 ± 0.000
Normal	1.000 ± 0.000	1.000 ± 0.000	1.000 ± 0.000	1.000 ± 0.000	1.000 ± 0.000

Abbreviations: PPV, positive predictive value; NPV, negative predictive value; F1, F1 score; HGSC, high-grade serous carcinoma; LGSC, low-grade serous carcinoma; CCC, clear-cell carcinoma; EC, endometrioid carcinoma; MC, mucinous carcinoma; RC, rare-type carcinoma

Supplementary Table S2. Slide-level classification performance of epithelial ovarian cancer subtypes in the cohorts at 20× magnification.

Subtype	Sensitivity	Specificity	PPV	NPV	F1
HGSC	0.367 ± 0.298	0.917 ± 0.032	0.333 ± 0.219	0.907 ± 0.035	0.342 ± 0.249
LGSC	0.350 ± 0.379	1.000 ± 0.000	0.600 ± 0.548	0.940 ± 0.032	0.423 ± 0.429
CCC	0.914 ± 0.078	0.960 ± 0.015	0.821 ± 0.066	0.983 ± 0.016	0.865 ± 0.067
EC	0.689 ± 0.093	0.943 ± 0.040	0.773 ± 0.158	0.922 ± 0.021	0.723 ± 0.097
MC	0.886 ± 0.064	0.902 ± 0.027	0.650 ± 0.037	0.976 ± 0.014	0.747 ± 0.006
RC	0.600 ± 0.224	0.969 ± 0.033	0.713 ± 0.278	0.959 ± 0.023	0.637 ± 0.200
Normal	1.000 ± 0.000	0.973 ± 0.019	0.813 ± 0.119	1.000 ± 0.000	0.893 ± 0.071

Abbreviations: PPV, positive predictive value; NPV, negative predictive value; F1, F1 score; HGSC, high-grade serous carcinoma; LGSC, low-grade serous carcinoma; CCC, clear-cell carcinoma; EC, endometrioid carcinoma; MC, mucinous carcinoma; RC, rare-type carcinoma

Supplementary Table S3. Patient-level classification performance of epithelial ovarian cancer subtypes in the cohorts at 40× magnification.

Subtype	Sensitivity	Specificity	PPV	NPV	F1
HGSC	0.933 ± 0.091	0.966 ± 0.042	0.871 ± 0.138	0.987 ± 0.017	0.893 ± 0.079
LGSC	0.783 ± 0.126	0.988 ± 0.016	0.900 ± 0.137	0.976 ± 0.014	0.831 ± 0.104
CCC	0.938 ± 0.085	1.000 ± 0.000	1.000 ± 0.000	0.987 ± 0.019	0.966 ± 0.046
EC	0.852 ± 0.102	0.973 ± 0.028	0.886 ± 0.120	0.966 ± 0.024	0.868 ± 0.104
MC	0.971 ± 0.064	0.973 ± 0.029	0.880 ± 0.137	0.993 ± 0.015	0.918 ± 0.088
RC	0.800 ± 0.209	0.987 ± 0.017	0.900 ± 0.137	0.975 ± 0.026	0.833 ± 0.156
Normal	1.000 ± 0.000	0.994 ± 0.014	0.960 ± 0.089	1.000 ± 0.000	0.978 ± 0.050

Abbreviations: PPV, positive predictive value; NPV, negative predictive value; F1, F1 score; HGSC, high-grade serous carcinoma; LGSC, low-grade serous carcinoma; CCC, clear-cell carcinoma; EC, endometrioid carcinoma; MC, mucinous carcinoma; RC, rare-type carcinoma

Supplementary Table S4. Patient-level classification performance of epithelial ovarian cancer subtypes in the cohorts at 20× magnification.

Subtype	Sensitivity	Specificity	PPV	NPV	F1
HGSC	0.813 ± 0.119	0.987 ± 0.018	0.920 ± 0.110	0.970 ± 0.021	0.861 ± 0.103
LGSC	0.493 ± 0.161	0.959 ± 0.025	0.693 ± 0.213	0.919 ± 0.019	0.562 ± 0.140
CCC	0.883 ± 0.162	0.994 ± 0.014	0.950 ± 0.112	0.988 ± 0.017	0.903 ± 0.092
EC	0.927 ± 0.101	0.927 ± 0.028	0.707 ± 0.097	0.987 ± 0.018	0.798 ± 0.076
MC	0.883 ± 0.162	0.970 ± 0.021	0.777 ± 0.137	0.987 ± 0.017	0.821 ± 0.128
RC	0.772 ± 0.080	0.979 ± 0.033	0.927 ± 0.104	0.940 ± 0.016	0.839 ± 0.068
Normal	0.717 ± 0.298	0.935 ± 0.057	0.668 ± 0.195	0.972 ± 0.028	0.626 ± 0.090

Abbreviations: PPV, positive predictive value; NPV, negative predictive value; F1, F1 score; HGSC, high-grade serous carcinoma; LGSC, low-grade serous carcinoma; CCC, clear-cell carcinoma; EC, endometrioid carcinoma; MC, mucinous carcinoma; RC, rare-type carcinoma
